# Statistical analysis of fractionation resistance by functional category and expression

**DOI:** 10.1186/s12864-017-3736-0

**Published:** 2017-05-24

**Authors:** Eric C. H. Chen, Annie Morin, Jean-Hugues Chauchat, David Sankoff

**Affiliations:** 1 Department of Biology, University of Ottawa, 30 Marie Curie, Ottawa, K1N 6N5 Canada; 2 Department of Computer Science, Université de Rennes 1, Rennes Cedex, 69676 France; 3 Laboratoire ERIC-Lyon2, Université de Lyon 2, Bron Cedex Cedex, 69676 France; 4 Department of Mathematics and Statistics, 585 King Edward, Ottawa, K1N 6N5 Canada

**Keywords:** Gene loss, Whole genome duplication, Gene ontology, Expression level, Angiosperms

## Abstract

**Background:**

The current literature establishes the importance of gene functional category and expression in promoting or suppressing duplicate gene loss after whole genome doubling in plants, a process known as fractionation. Inspired by studies that have reported gene expression to be the dominating factor in preventing duplicate gene loss, we analyzed the relative effect of functional category and expression.

**Methods:**

We use multivariate methods to study data sets on gene retention, function and expression in rosids and asterids to estimate effects and assess their interaction.

**Results:**

Our results suggest that the effect on duplicate gene retention fractionation by functional category and expression are independent and have no statistical interaction.

**Conclusion:**

In plants, functional category is the more dominant factor in explaining duplicate gene loss.

## Background

**Fig. 1:**
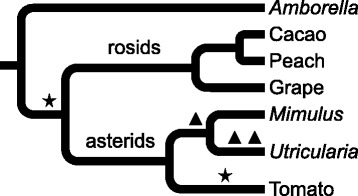
Whole genome duplication history. Star symbols mean whole genome triplication events while triangle symbols are duplication events [3, 20, 30]. Phylogeny branch lengths not to scale

The proliferation and the advancement of tools for genetic analysis changed the understanding of the role of polyploidy in evolution [1]. Polyploidy, which can result from whole genome duplication events of doubling or tripling of the genome, is now considered to be a recurrent and frequent theme in plant evolution. Virtually all land plants have a polyploid ancestor [2–5] with many lineages having additional rounds of whole genome duplication events (Fig. 1). These special events in evolutionary history have been linked to increased morphological and genetic diversity [6, 7].

After whole genome duplication events there is massive duplicate gene loss, a process known as fractionation. Duplicate genes from whole genome duplications are sensitive to pseudogenization and excision of chromosomal fragments. Notably, fractionation continues even after the polyploid species has been rediploidized. Models such as the Gene Balance Hypothesis [8] and the Gene Dosage Hypothesis [9, 10] attempt to explain the pattern of these duplicate gene losses [11].

**Fig. 2:**
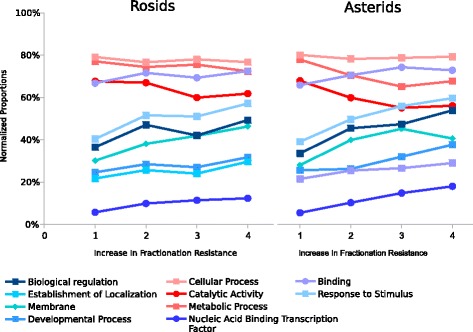
Based on retention of paralogs resulting from ancient polyploidization in three rosids and three asterids. Retained genes identified in homeologous syntenic blocks detected by SynMap [17, 18]. “Increase in fractionation resistance” ranges from 1 (singleton in all three species) to 4 (three paralogs retained in all species). “Normalized proportions” measures how many of the gene paralogy groups with a given fractionation resistance are annotated by a specific Gene Ontology (GO) term. E.g., in the rosids, 80% of the paralogy groups with fractionation resistance 1 are annotated with the GO term “Cellular Process”. From [14], Figure 3

The Gene Balance Hypothesis argues that the need to maintain stoichiometry ratio between important gene products results in the maintenance of these duplicate genes. In this model, duplicate regulatory genes and duplicate genes responding to stimulus are expected to be maintained at a greater rate due to gene product interactions. Gene products that do not need to interact with other gene products to maintain a delicate balance, such as many metabolic and enzymatic genes which interacts with metabolites such as food, sugar, and fat, are expected to be lost at a greater rate. We have verified these general expectations in previous work [12–14] as documented in Fig. 2.

A striking example of gene balance is provided by the preferential retention of circadian clock genes after the whole genome triplication event in the history of *Brassica rapa* [15]. The regulation of these genes in plants is assured by stoichiometric negative feedback loops. These clock genes, as a whole, are preferentially retained compared to other core eukaryotic genes or to neighbouring genes flanking the clock genes.

**Fig. 3:**
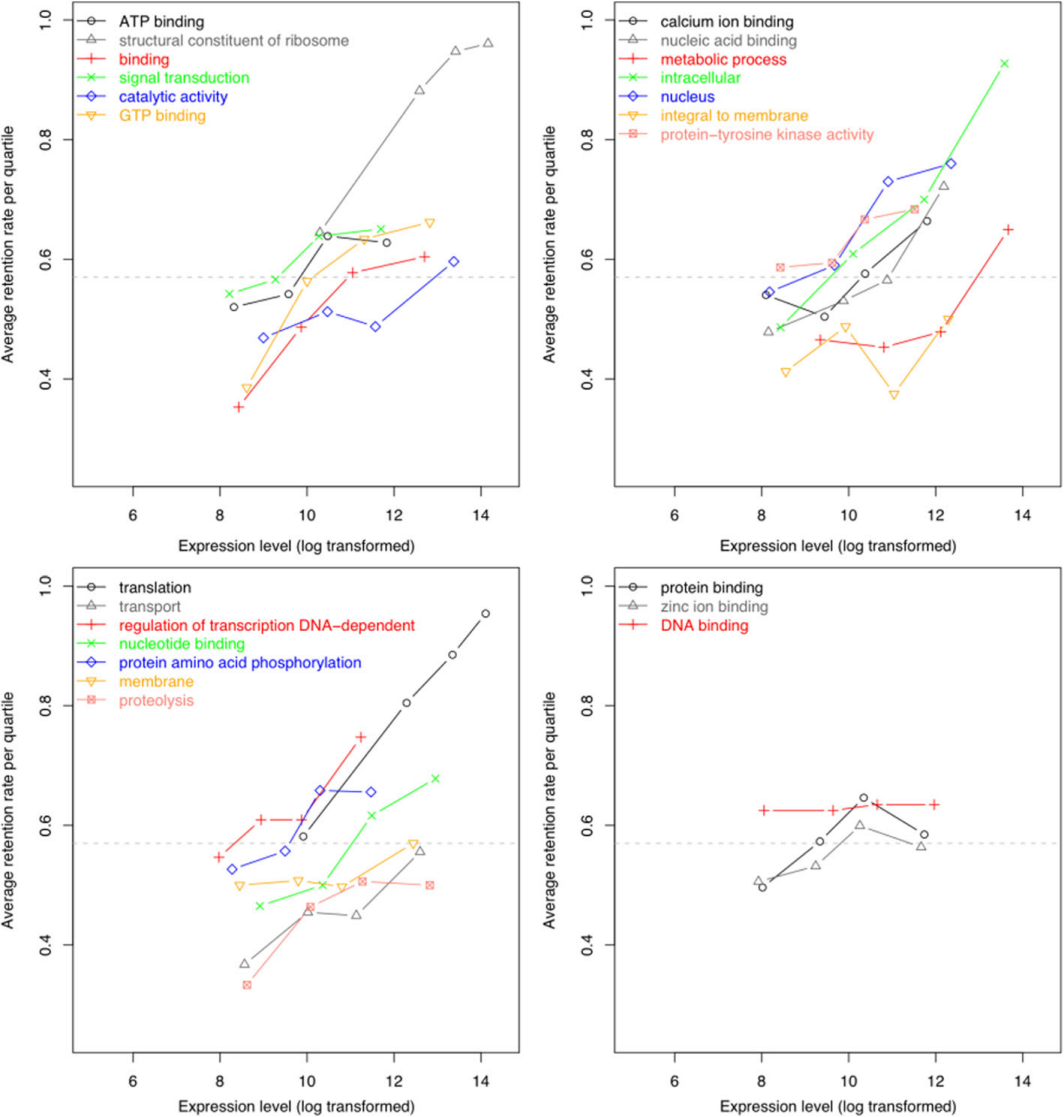
The *Paramecium* genes are filtered by GO terms before putting inside the expression bins. The Y-axis describes the retention rate of genes inside the expression bins. From [16], Figure S3

The competing model, the Gene Dosage Hypothesis, argues that important genes are simply more likely to be kept, and because of how biologically expensive it is to maintain high expression levels, high gene expression level is a good indicator that the gene is important. Prior to the WGD, loss of these genes would entail significant loss of fitness. After WGD, the organism has reached a new normal, with twice the previous activity, and disproportionate loss of these expensive gene via fractionation would also incur a decrease of fitness. Therefore, duplicate genes with high expression levels will be maintained in duplicate. In this model, gene function is still the driving force to maintain these duplicates, but high level general functional categories, such as the above-mentioned metabolic, enzymatic, regulatory, and response patterns, are too general to be of use in predicting duplicate gene retention. Gout et al. [16] reported, in *Paramecium*, that high expressing genes are maintained in duplicate more than low expressing genes. Controlling for different functional categories having different expression levels does not change this result (Fig. 3). In [14], we also reported that duplicate genes are more likely to be maintained as duplicates if they have high expression levels, regardless of their functional categories. However, our results showed the effect of gene expression on maintaining duplicate gene after whole genome duplication events is much less pronounced than in the *Paramecium* study.

Both the Gene Balance Hypothesis and the Gene Dosage Hypothesis are needed because each model explains observations that the other model can not fully explain. However, teasing apart the relative importance of those factors require rigorous multivariate analysis. This what we undertake in the paper, and despite the intuitive appeal of the Gene Dosage Hypothesis, we find that gene functional category is far more explanatory of variable retention rate than gene expression.

## Methods

### Data

We construct gene families based on the sequence similarity and the conserved gene order between extant species using CoGe [17, 18]. These gene families are pruned into smaller units that are linked by the whole genome duplication in the ancestor using the “Orthologs for Multiple Genomes” program [19]. Detailed flowcharts and parameters for generating gene families have been presented previously [12, 13].

The species grape [20], peach [21] and cacao [22] form the rosid data set. These species can trace their last common ancestor to the period after the divergence of the asterids, following the core eudicot hexaploid about 120 million years ago [3]. There are no additional rounds of whole genome duplication in the evolutionary paths leading to the these present-day species [20–22]. Therefore, whole genome comparative analysis of the rosid data set offers insights on the effects of fractionation over long period of time.

The asterid data set provides a different viewpoint of the fractionation process compared to the rosid data set. The last common asterid ancestor diverged five to ten million years after the hexaploid core eudicot ancestor. This early divergence means the fractionation process after the hexaploid ancestor of the asterid data set is mostly independent from the fractionation process in the species of the rosid data set. Furthermore, the species of the asterid data set, which consists of extant species tomato [23], *Mimulus* [24], and *Utricularia* [25], have additional rounds of whole genome duplication [3].

The asterid data set addresses two potential concerns. The first concern is whether the results of the rosid data set represent a general effect or a clade-specific trend. The second concern is whether the additional rounds of whole genome duplication introduce a different pattern compared to single ancient whole genome duplication event. Thus far the fractionation pattern of genomes of the datasets is consistent with the literature and appears to be general [11, 13].

For the expression analysis, we use grape to represent the rosids and tomato to represent the asterids. High quality RNA-seq expression data, already normalized and organ-specific, are available for both species [23, 26]. Since a gene’s function may be relevant to specific tissues only, for each gene, we use the highest expression level it displays across all organs to represent its expression score.

### Retention indices

We use retention indices to measure how fractionation resistant or prone gene families are. The retention index of each gene family is calculated by counting in how many species the genes is still maintained in duplicate. For example, if a gene family of the rosid data set is maintained as duplicates only in grapes, then the retention index of that gene family is one. Since there are three species in both the rosid data set and the asterid data set, retention indices range between zero (gene set reduced to singletons in all species) and three (gene maintained as duplicates in all species).

**Fig. 4:**
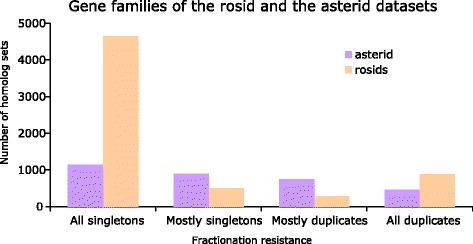
Number of gene families of in each fractionation resistance categories. “All singletons” have retention index of zero, “mostly singletons” have retention index of one, “mostly duplicates” have retention index of two, and “all duplicates” have retention index of three

Figure 4 summarizes how many gene families are in each retention category based on each gene family’s retention index. For rosids, a much larger proportion of gene families have become singletons. While the “all singletons” (retention index of zero) category also contain the highest number of gene families in asterids, the families are more evenly distributed among the retention categories.

### Expression

For the expression analysis, we use individual genes instead of gene families, for two reasons. The first reason is that genes in duplicate families have varying gene expressions that may differ by orders of magnitude. The skewness of the data prevents us from using averages. Second, we cannot just take the highest expressing gene in the gene family in the same way as we chose the organ with the highest expression to represent the gene’s score. This is to avoid the artifact that the more genes a gene family has, the higher the expression of the gene family will be by virtue of having more chance to include a high expressing gene.

We also bin gene expression data into two groups, HighExp and LowExp, as an additional normalization step. Genes of the HighExp group have expression levels greater or equal than the median gene expression level of the particular functional category. The LowExp group contains genes that have expression levels lower than the median gene expression level of the particular functional category.

### Annotations

**Fig. 5:**
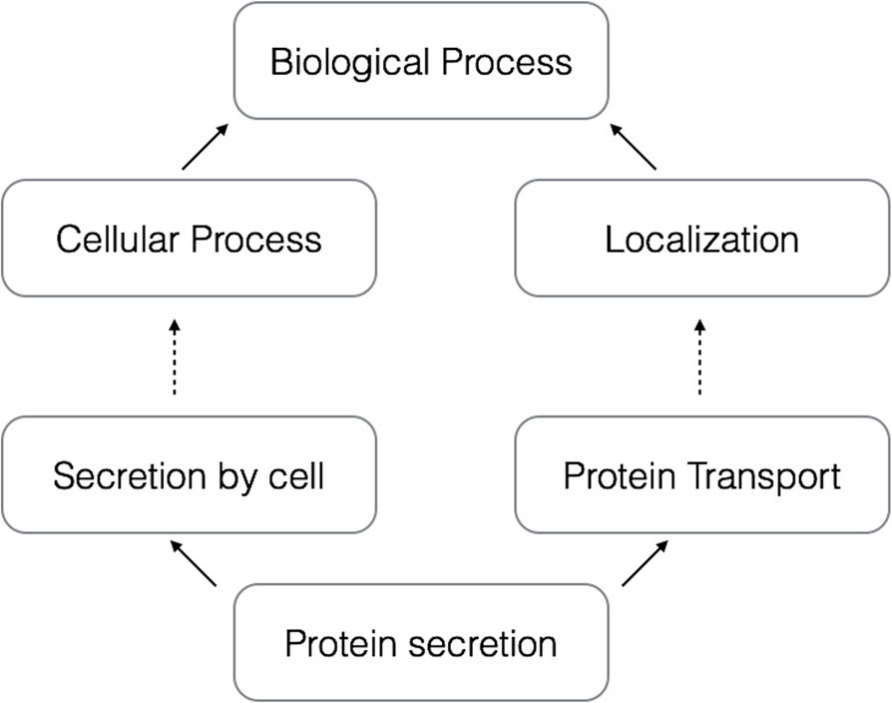
Example of nested structure of GO terms. Starting at a low-level GO term “protein secretion”, it is inherited by two higher GO terms “secretion by cell” and “protein transport”. After a few more levels of GO terms (represented by dashed lines), the starting GO term is now inheriting two high level terms “cellular process” and “localization”. These high level terms are then linked to the root term, “biological process”. There are three root terms in gene ontology, they are “biological process”, “cellular component”, and “molecular function” [27]

We use GO [27] terms to classify gene families into functional categories via Blast2GO [28]. GO terms are nested within each other to provide different resolution of annotation (Fig. 5). We call GO terms that are close to the one of the three “root terms” “high level terms”. These high level terms describe general functional categories. As a result, a particular gene may be annotated with multiple high level terms as shown in Fig. 5.

We designate three high levels of GO functional categories (Fig. 5) that we previously found to have the highest effect on fractionation [13, 14]. The first category is “Metabolic process (Z1)”, one of the most fractionation-prone. The second category is “Enzyme class (Z2)”. It is also highly fractionation-prone but it includes enzymes involved in signalling pathways so the category as a whole may show increased retention compared to Z1. The third category is “Regulation and Response” (Z3). This is composed of two most fractionation-resistant GO categories. These three high level GO functional categories cover two of the three GO distinct domains: “biological process” (Z1 and Z3) and “molecular function” (Z2).

Each high level functional categories is further divided into six low-level GO categories to represent more specific and biologically distinct functions. GO terms “secondary metabolic process”, “lipid biosynthetic process”, “steroid metabolic process”, “nucleobase-compound containing metabolic process”, “carbohydrate metabolic process”, and “protein metabolic process” represents Z1. These six metabolic GO terms are representative of diverse metabolic processes. GO terms “transferase activity”, “oxidoreductase activity”, “hydrolase activity”, “ligase activity”, “lyase activity”, and “isomerase activity”, the six major enzyme classes, represent Z2.

GO terms “regulation of metabolic process”, “nucleic acid transcription factor activity”, “signal transduction”, “response to hormone”, “response to temperature”, and “response to stress” represent Z3. This is a combination of two highly fractionation-resistant functional categories in “biological regulation” and “response to stimulus” [13] so that there are six low level and biologically distinct GO terms in each high level functional categories (Table 1).

**Table 1:** GO terms and number of genes

				Tomato	Grape
Metabolic Process (Z1)	Z11	GO.0008610	lipid biosynthetic process	286	397
	Z12	GO.0008202	steroid metabolic process	54	75
	Z13	GO.0006139	nucleobase containing compound metabolic process	655	1055
	Z14	GO.0005975	carbohydrate metabolic process	575	810
	Z15	GO.0019538	protein metabolic process	1109	1389
	Z16	GO.0019748	secondary metabolic process	131	214
Enzyme Class (Z2)	Z21	GO.0016740	transferase activity	962	1227
	Z22	GO.0016491	oxidoreductase activity	529	693
	Z23	GO.0016787	hydrolase activity	878	1254
	Z24	GO.0016874	ligase activity	177	246
	Z25	GO.0016829	lyase activity	119	152
	Z26	GO.0016853	isomerase activity	67	131
Regulation Response (Z3)	Z31	GO.0019222	regulation of metabolic process	965	1043
	Z32	GO.0001071	nucleic acid binding transcription factor activity	403	324
	Z33	GO.0007165	signal transduction	550	573
	Z34	GO.0009725	response to hormone	492	464
	Z35	GO.0009266	response to temperature stimulus	291	284
	Z36	GO.0006950	response to stress	1032	1301

## Results and discussion

From our previous results [13, 14], we predict Z1 to be the most fractionation-prone, closely followed by Z2, and then Z3.

**Fig. 6:**
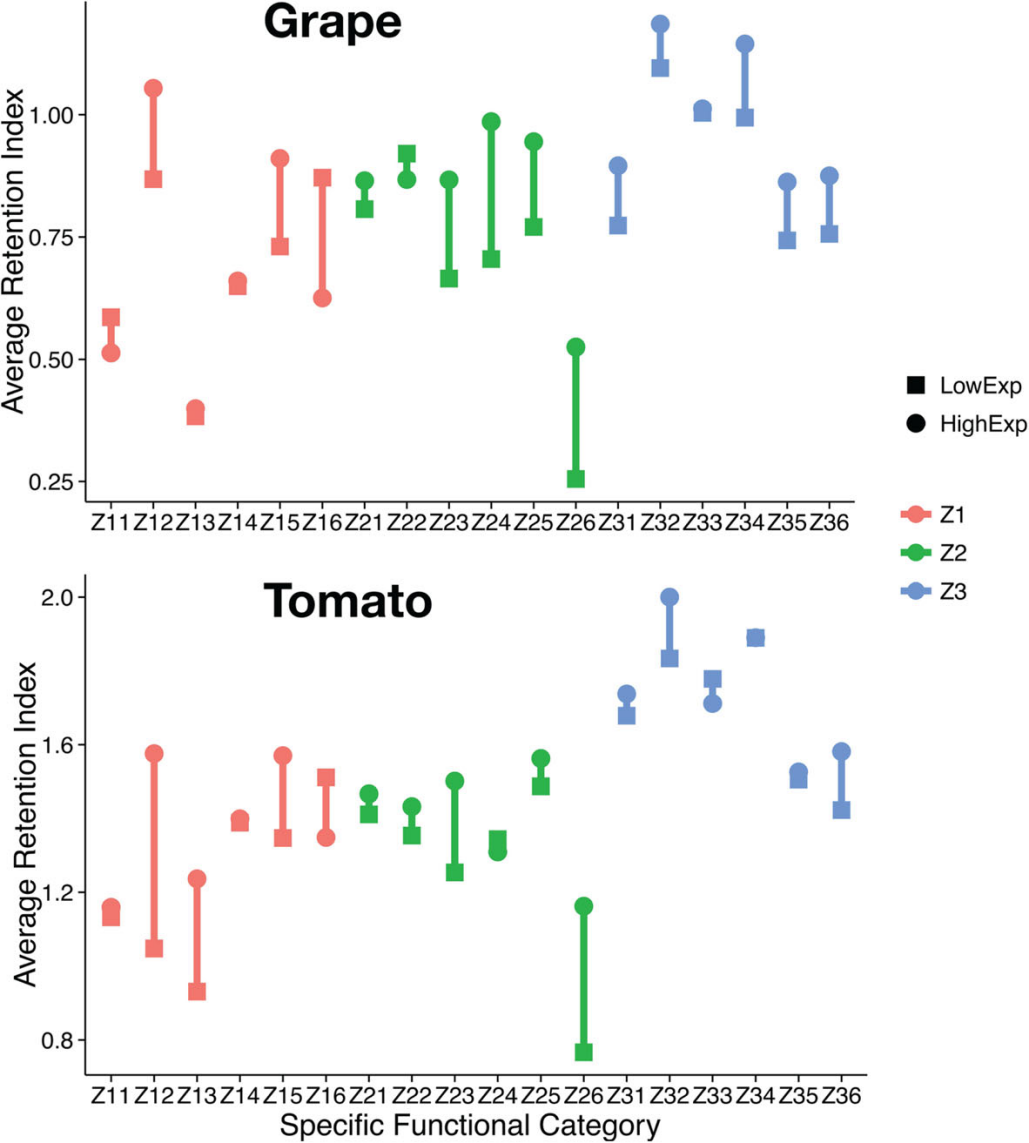
Summary of average retentions indices in grape and tomato. Each functional category has two data points: average retention index under low expression (LowExp) and average retention index under high expression index (HighExp)

The inherently different gene count for different functions (Table 1) means the categories are not balanced as would be required for ANOVA. We sidestep the issue by using the average retention index of each functional category instead of the raw count. This strategy comes at the expense of statistical power since we are now left with just two data points for each low-level functional category. Still, Fig. 6 shows the expected result of high expression correlating with high fractionation resistance.

Figure 6 is a visual representation of what the average retention indices are for each functional category. This result is consistent with our prediction that genes of Z3 are more fractionation-resistant than gene of Z2 and Z1.

**Fig. 7:**
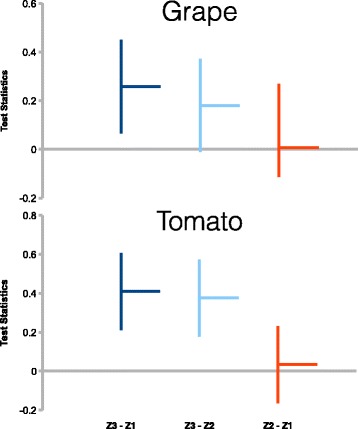
Tukey’s honest significant difference test. The horizontal bar indicate the Tukey test statistics (which include corrections for multiple comparisons) of the estimated difference between labelled categories. The vertical lines indicate the 95% confidence interval. In both grape and tomato, category Z2 and Z1, in red, are not significantly different from each other. In grape, category Z3 and Z2, in light blue is not highly significant (adjusted p-value is 0.06887)

This is further reinforced in Fig. 7. This supports our prediction that genes of Z3 are more fractionation-resistant than Z1 and Z2. In grape, the adjusted p-value for the statistical test of the difference between Z3 and Z2 is only marginally significant, likely due to insufficient data. That the difference is real is bolstered by the clear difference between Z3 and Z2 in tomato.

Figure 7 also shows that in grape, the difference between fractionation-resistant Z3 and fractionation prone Z1 and Z2 are smaller than the difference in tomato. A reason for this observation being that gene families that are singletons in all three species of the rosid data set constitute a far more higher proportion than in the asterid data set, so even the fractionation-resistant functional category contain many singleton gene families.

The ANOVA table (Table 2) answers the main objective of the paper: which of Gene Balance Hypothesis and Gene Dosage impact duplicate gene retention more? We answer this by calculating whether functional categories or expression levels have the bigger effect size in the two-way ANOVA. In the table, the effect size, measured in partial eta squared, supports the conjecture in the Chen et al. paper [14] that functional category carries more weight in determining retention indices than expression levels. The table also shows that while functional categories strongly affect average retention indices, the effect that expression levels have on average retention indices are no longer significant.

**Table 2:** ANOVA table on balanced grape and tomato data

Grape Anova Table (Type II tests)						
	Partial etaˆ2	Sum Sq	Df	F value	Pr(>F)	
GOf	0.9071	36.193	3	97.64771	<1e-15	***
ExpQ	0.06236	0.121	1	1.9953	0.1681	
GOf:ExpQ	0.02797	0.017	2	0.4317	0.6534	
Residuals		1.0918	30			
Tomato Anova Table (Type II tests)						
GOf	0.96865	36.193	3	308.9591	<2e-16	***
ExpQ	0.0937	0.121	1	3.1016	0.08841	.
GOf:ExpQ	0.01395	0.017	2	0.2121	0.81005	
Residuals		1.171	30			

^*^GOf is the High level functional category. ExpQ is the expression category

## Conclusion

Expression has been suggested to be the most important factor in determining duplicate retention after whole genome duplication events [16]. Our results suggest otherwise, that functional category is the more dominant factor of the two. Furthermore, our results in Table 2 suggests that there is no interaction between functional category and expression level.

We expect the result presented here to be present in other flowering plant lineages as well, given how both the rosid dataset and the asterid dataset show a consistent trend. Also, our previous analyses on fractionation resistance [13, 14] show these retention trends to be consistent across different lineages, giving us more confidence in this prediction.

Going forward, we want to further explore the role of expression on fractionation. One direction is to explore the different types of expression. Some genes are only expressed in certain tissues or at certain developmental stages, such as the development of flowers, or genes that have organ specific expression pattern, or genes that are always on but fluctuate depending on the situation. Different expression pattern may have different fractionation tendencies.

Another direction is to expand the analysis to other genes that are currently not part of the analysis. One particular analysis for future work is the relationship between retained duplicates and the nearby genes. Retained duplicates are reported to have an effect on the distribution of genes with copy number variation in humans [29]. We can explore if similar effects are also present in plants.

In summary, we have evidence to suggest that functional categories plays a more important than gene expression levels in duplicate gene retention after whole genome duplication. There are many challenges and possibilities that can build upon this work to better explain the mechanisms and the effects of the fractionation process.
